# Personalized Perioperative Opioid Strategies in Children: Focus on Methadone, Pharmacogenomics and Prevention of Persistent Postoperative Opioid Use

**DOI:** 10.3390/children12121660

**Published:** 2025-12-07

**Authors:** Hamsa Priya Bhuchakra, Sennaraj Balasubramanian, Alivia G. Nair, Isabella Marcos, Victoria Chen Falconett, Dominic Falcon, Ayesha Abdul Bari, Senthilkumar Sadhasivam

**Affiliations:** 1 Department of Anesthesiology and Perioperative Medicine, UPMC Children’s Hospital of Pittsburgh, University of Pittsburgh, Pittsburgh, PA 15224, USA; priyah@upmc.edu (H.P.B.); agn31@pitt.edu (A.G.N.); imarcos@friars.providence.edu (I.M.); falconettvc@upmc.edu (V.C.F.); dfalcon27@jcu.edu (D.F.); 2 Apollo Institute of Medical Sciences and Research, Hyderabad 500096, India; ayeshaabdulbari@gmail.com; 3 Department of Anesthesiology, Washington University School of Medicine in Saint Louis, Saint Louis, MO 63110, USA; bsennaraj@wustl.edu; 4 University of Pittsburgh, Pittsburgh, PA 15260, USA; 5 Providence College, Providence, RI 02918, USA; 6 West Virginia University, Morgantown, WV 26506, USA; 7 John Carroll University, University Heights, OH 44118, USA

**Keywords:** pediatric perioperative care, persistent postoperative opioid use, methadone, pharmacogenomics, personalized pain management, opioid stewardship, multimodal analgesia, central sensitization

## Abstract

Persistent postoperative opioid use (PPOU) is an emerging challenge in pediatric perioperative care, with rates as high as 4.7% in opioid-naive adolescents. Despite advances in multimodal analgesia, current protocols often fail to prevent long-term opioid exposure, particularly after high-risk surgeries such as spinal fusions. While multiple strategies exist to reduce PPOU in children, including regional anesthesia and non-opioid analgesics, this review specifically focuses on methadone and pharmacogenomic-guided opioid prescribing as promising approaches. Methadone, a long-acting opioid with mu-opioid agonism, NMDA antagonism, and monoamine reuptake inhibition, has shown encouraging outcomes in adult and emerging pediatric studies but remains underutilized due to concerns over safety, variability, and familiarity. This narrative review explores the intersection of methadone pharmacology, pharmacogenomic (PGx)-guided opioid prescribing, and their potential to reduce PPOU and optimize perioperative pain control in children. We examine methadone’s unique pharmacokinetic profile, extended half-life, and ability to reduce central sensitization and opioid tolerance. Data from pediatric trials in cardiac, spinal, and major abdominal surgeries are reviewed, highlighting methadone’s potential to lower total opioid use, stabilize postoperative pain trajectories, and improve recovery. The review also discusses the role of PGx testing, particularly CYP2D6, CYP3A4, UGT2B7, and OPRM1 variants, in tailoring methadone dosing to individual metabolic profiles, reducing adverse effects, and improving analgesic efficacy. There are no well accepted generalizable perioperative methadone dose, number of doses and dosing intervals due to limited large multicenter studies in children. We outline challenges, including QTc prolongation, dosing variability, lack of pediatric-specific PGx guidelines, and ethical considerations around genetic testing in minors. The review calls for multidisciplinary perioperative teams, expanded PGx implementation, and real-world data from registries and AI-integrated models to support precision opioid strategies. Preventing PPOU in children is critical. Integration of methadone-based multimodal analgesia in high-risk painful in-patient procedures and future integration of PGx represent positive steps toward personalized, effective, and safer pain management in pediatric surgical patients, an urgent need as opioid stewardship becomes a clinical and public health imperative.

## 1. Introduction

Opioids are often used in the immediate postoperative period to manage acute pain. However, concerns exist in adolescents, especially orthopedic/abdominal surgery, due to risks of overprescription and long-term dependence. One specific consequence is Persistent Postoperative Opioid Use (PPOU), commonly defined as filling one or more opioid prescriptions between 90 and 180 days after surgery. According to recent data, PPOU occurs in up to 7% of opioid-naive pediatric patients, with reported rates of 4.7 per 100 adolescents and young adults, and the incidence has been steadily rising since the late 1990s [1,2,3]. These risks are amplified in adolescents, who are developmentally more vulnerable to addiction and are frequently prescribed opioids for procedures associated with moderate to severe postoperative pain [1,3].

This mirrors broader opioid misuse trends, highlighting the limits of current multimodal protocols in preventing PPOU [4,5,6]. While these protocols have demonstrated success in reducing in-hospital opioid use, few studies have extended their follow-up beyond discharge, leaving the long-term impact on opioid dependence unclear. This gap underscores the need for novel strategies to more effectively prevent PPOU. Other strategies to reduce PPOU include multimodal analgesia with non-opioid medications (e.g., NSAIDs, acetaminophen) and regional anesthesia; however, this review focuses on methadone and pharmacogenomic-guided opioid prescribing [7].

One promising avenue for addressing this issue is methadone, a long-acting opioid with unique pharmacologic properties. It was first synthesized in 1938 and has been used to treat opioid dependence as well as chronic and acute surgical pain. Methadone acts as an NMDA receptor antagonist, has a long half-life providing sustained analgesia, and produces less euphoria than many other opioids, which may help reduce the risk of persistent use and tolerance development [5,8,9]. Despite its clinical and notably lower abuse potential and less sedation compared to morphine, methadone remains underutilized. This is due to the fear of increased QTc, unpredictable kinetics, lack of training and unfamiliarity among pediatric providers, and large interpatient variability [9,10].

Methadone’s risk of severe respiratory depression necessitates considerations and analysis of its pharmacokinetics. Its metabolism differs by stereoisomer and CYP activity. Several CYP enzymes, including CYP2B6, CYP2D6, CYP2C19, CYP2C9, and CYP3A4, have been found to play key roles in methadone metabolism [11,12,13]. CYP2B6 is the primary metabolizer of S-methadone, and CYP3A4 is the primary metabolizer of R-methadone. These findings have led to an increased interest in pharmacogenomics (PGx), which tailors drug therapy based on genetic differences in enzymes such as CYP2D6 and CYP3A4 [11]. Since CYP2D6 activity varies greatly between individuals due to genetic polymorphisms, understanding a patient’s genotype could optimize methadone use. Optimization of methadone could maximize efficacy while minimizing risks such as respiratory depression and reducing the likelihood of PPOU. This personalized strategy holds promise for mitigating PPOU and optimizing pain management outcomes [11,14,15].

Hence, this narrative review explores how the integration of pharmacogenomics with methadone can form the basis of precision opioid strategies that address persistent postoperative opioid use in pediatric surgical patients.

## 2. Persistent Postoperative Opioid Use (PPOU) in Pediatrics

Despite ongoing efforts to improve perioperative pain management, persistent postoperative opioid use (PPOU) remains a growing concern in pediatric surgery [16,17,18]. Understanding the risk factors and variability in PPOU is critical for developing precision-based pain management strategies in children.

In a retrospective cohort of 88,637 children aged 0 to 18 years, prolonged use was defined as opioid prescriptions for at least 90 days and persistent use as at least 180 days postoperatively. Rates were 0.7% and 0.1%, respectively [19]. Risk was highest after spinal fusion (1.6%), major abdominal surgery (1.2%), and orthopedic procedures (0.5%), compared with less than 0.1% for ENT and dental procedures. Although the percentages appear low, the implications are significant given the vulnerability of the developing brain. Adolescents were disproportionately affected, making up 72% of persistent users despite representing only 54% of the cohort. In a multicenter study of 682 patients, adolescents aged 13 to 18 required 35% more opioids postoperatively than children aged 2 to 12 (*p* < 0.01) [20], reflecting developmental differences in physiology and behavior. Additional analyses suggest adolescence is a high-risk period for misuse due to peer influence, impulsivity, and limited awareness of opioid consequences [21]. Younger children also remain vulnerable when exposed for prolonged periods during recovery.

Chronic postsurgical pain in children is another key long-term outcome linked to acute pain and early opioid use. Reported prevalence ranges from 2 to 100% across surgical types, with spinal fusion showing the highest rates [22]. A pediatric meta-analysis found a median prevalence of 20% at 12 months post-surgery [23]. These findings underscore the importance of early, individualized analgesic strategies to reduce both acute pain and long-term opioid risk.

Procedure type is a well-established predictor of PPOU. In a retrospective analysis of 36,177 adults, thoracic and orthopedic surgeries were most associated with chronic use (OR 1.6, 95% CI: 1.2–2.0; *p* < 0.001) [24]. Pediatric-specific studies remain limited, but adult data can offer early insights because of similar surgical complexity and pain trajectories. Available pediatric evidence, including a meta-analysis of more than 85,000 cases, shows that thoracic (RR 2.4), orthopedic (RR 2.1), and major abdominal surgeries carry the highest PPOU risk [25]. These findings highlight the urgent need for additional pediatric research.

Regional anesthesia plays a central role in reducing postoperative opioid exposure. Techniques such as epidural, paravertebral, erector spinae, and peripheral nerve blocks have been shown to lower opioid requirements by 30% to 60% in children undergoing high-risk procedures [25,26,27]. In adolescents, minimizing early opioid exposure is especially important given behavioral and developmental vulnerabilities. Large multicenter studies confirm that regional techniques are generally safe, with no permanent neurologic deficits (95% CI 0–0.4 per 10,000), a low incidence of transient neurologic symptoms (2.4 per 10,000; 95% CI 1.6–3.6 per 10,000), and rare episodes of severe systemic toxicity (0.76 per 10,000; 95% CI 0.3–1.6 per 10,000), most of which occurred in infants. Benign catheter-related issues were the most frequent complications (4%) [28]. Despite these established benefits, regional anesthesia is often underrepresented in PPOU analyses because of variability in block placement, timing, provider expertise, and institutional protocols [28].

Dosing inconsistencies further amplifies risk. A cross-sectional review of 317 hospitalized infants across NICUs found greater than 25% deviation in morphine milligram equivalency (MME) standardization, increasing the likelihood of overexposure [29]. Such variability may disrupt endogenous opioid pathways, fostering tolerance, and later misuse. Additional clinical risk factors include preoperative opioid exposure (OR 5.22; 95% CI: 4.80–5.68), female sex (OR 1.22; 95% CI: 1.10–1.36), and musculoskeletal surgery (OR 1.78; 95% CI: 1.52–2.09) [30]. Psychiatric comorbidities such as depression, anxiety, and ADHD may elevate risk through altered pain perception, impaired coping, and increased baseline opioid exposure. Chronic pain conditions like juvenile idiopathic arthritis or sickle cell disease also increase susceptibility, particularly when opioids are used preoperatively.

The interplay between anesthesia modality, genetic predispositions, and mismatched pain management may further contribute to misuse [16]. Although pediatric data on OPRM1 and COMT variants are limited, their biologic relevance supports investigation. A national prescribing review across 43 USA hospitals revealed substantial variability in infant opioid dosing (MME/day range: 0.02–0.36 mg/kg, *p* < 0.001) [31], underscoring the urgent need for standardized guidelines and stewardship interventions.

Methadone has emerged as a promising adjunct in pediatric multimodal analgesia. In a prospective cohort study of 102 children undergoing congenital heart surgery, intraoperative methadone halved postoperative opioid requirements (mean MME: 0.84 vs. 1.65, *p* = 0.01) and improved extubation time [17]. Likewise, scheduled methadone dosing in pediatric spinal fusion patients reduced both opioid consumption and pain scores, with a 23% lower cumulative requirement (*p* = 0.03) [32]. A high-quality randomized controlled trial (RCT) involving 65 adolescents undergoing spinal fusion found that methadone-based analgesia reduced opioid use by 40% (*p* < 0.01) and improved early ambulation [33].

A pediatric scoping review of 19 studies emphasized the 90-day postoperative period as a clinically meaningful threshold for PPOU, highlighting the importance of early interventions, including methadone, in reducing long-term opioid dependence [34]. A systematic review of pediatric palliative care cases also supported methadone’s favorable pharmacokinetics (NMDA antagonism, long half-life), while cautioning that genetic variability in CYP2D6 and CYP3A4 may increase the risk of over-or under-treatment without guidance [35]. These findings reinforce the potential of integrating pharmacogenomic insights, particularly CYP2D6, COMT, and OPRM1, into pediatric perioperative opioid use frameworks.

Tailoring opioid strategies by surgical complexity is increasingly supported. In a matched cohort study comparing methadone-based anesthesia with epidural analgesia in pediatric Nuss procedures, methadone was associated with shorter ICU stays (2.1 vs. 3.4 days, *p* = 0.02) and 38% lower opioid use [18], though implementation requires careful titration to avoid oversedation.

Despite growing interest, research on PPOU in pediatrics remains limited. Much of the existing evidence is extrapolated from adult populations, and pediatric-specific RCTs are sparse. Inconsistencies in definitions, regional prescribing practices, and MME conversions hinder standardization and comparison. Furthermore, while pharmacogenomic predictors like CYP2D6, OPRM1, and COMT are biologically plausible, they remain underutilized in clinical pediatric settings. Addressing these limitations will require longitudinal, age-specific research, improved pharmacokinetic modeling, and the adoption of genotype-informed prescribing practices [35].

Pediatric PPOU is a multifactorial issue shaped by individual characteristics (e.g., age, sex, mood disorders), procedural variables (e.g., surgery type), and pharmacologic approaches. Methadone has shown encouraging results as part of a multimodal strategy, and pharmacogenomics may enhance personalization of therapy. Ultimately, progress in perioperative opioid stewardship will depend on implementing standardized protocols, early identification of high-risk patients, and integrating genetic and clinical factors into precision-based frameworks for pediatric pain management [35].

## 3. Methadone: Pharmacologic Profile and Peri-Operative Promise

### 3.1. Methadone: Mechanism of Action

Methadone is a synthetic opioid with a distinct pharmacologic profile that allows it to address pain through multiple mechanisms. Methadone’s dual action in both analgesia and addiction treatment reflects its versatility [16]. Its primary analgesic action is through activation of mu-opioid receptors (MORs) in the brain and spinal cord, which reduces the release of excitatory neurotransmitters like glutamate and substance P in transmitting pain signals. At standard analgesic doses (0.1–0.2 mg/kg), methadone can achieve 85–90% MOR occupancy and inhibit neurotransmitter release in dorsal horn synapses by approximately 70–90%, correlating with significant reductions in pain perception [26]. What sets methadone apart from conventional opioids like morphine and fentanyl is its ability to block NMDA receptors and inhibit the reuptake of serotonin and norepinephrine. Unlike drugs that act solely through MOR agonism, methadone’s NMDA antagonism has been shown to reduce central sensitization and opioid-induced hyperalgesia, particularly at plasma concentrations as low as 100–400 ng/mL [27]. According to Durrani and Bansal (2024), this broader activity makes methadone effective in treating not only nociceptive but also neuropathic pain, offering a more versatile and targeted approach to analgesia than traditional opioids alone [27].

### 3.2. Long Half-Life and Multimodal Effects

This multimodal activity is supported by methadone’s long half-life, ranging from 8h to 59 h in adults and up to 90 h in pediatric patients, compared to 2–4 h for morphine and 3–12 h for fentanyl. This allows methadone to maintain therapeutic levels longer, sustaining NMDA antagonism and prolonged analgesia [7,27,36]. Ramaiah and Kharasch (2024) described an RCT in adult surgical patients where intraoperative methadone was integrated into recovery protocols. This approach reduced postoperative pain scores by approximately 2 points on a 0–10 numeric rating scale and decreased opioid consumption by up to 40% in MME compared to standard opioid regimens [37]. These findings highlight methadone’s role in improving analgesia while minimizing the need for additional opioids postoperatively [37]. Martin et al. (2018) conducted another RCT in adolescents undergoing posterior spinal fusion (*n* = 59), finding that patients who received intraoperative methadone had significantly lower pain scores, averaging 3.4 vs. 5.1 on a 0–10 scale at 12 h post-operatively, and required 30% less opioid rescue medication over the first 24 h compared to the standard care group [36]. Similarly, Chen et al. (2023) analyzed pediatric postoperative data from a retrospective cohort and found that methadone’s extended duration led to more consistent analgesia, with fewer pain score fluctuations (mean variation of ±0.6) compared to short-acting opioids (±1.5), and a 25% reduction in total opioid use across the first 48 h [7].

## 4. Reduced Central Sensitization and Lower Opioid Use

By blocking NMDA receptors, methadone reduces central sensitization and delays the development of opioid tolerance, which are two key components of escalating opioid requirements after surgery. As a result, methadone use may help reduce postoperative opioid consumption. Nguyen et al. conducted a retrospective study in 56 pediatric patients undergoing cardiothoracic surgery, comparing intraoperative methadone to caudal morphine. Patients in the methadone group had significantly lower postoperative pain scores, averaging 2.1 vs. 4.5 on a 0–10 scale during the first 12 h after surgery [38]. This is echoed by Blasiole et al., who retrospectively studied children undergoing cardiac surgery. They found that those who received intraoperative methadone had significantly lower opioid use on the day of surgery (median 0.3 vs. 0.5 MME/kg, *p* = 0.005), with no increases in outcomes like naloxone use or reintubation. The cohort included 287 pediatric patients aged 1 month to 18 years undergoing various cardiac surgeries, such as ventricular septal defect repair, tetralogy of Fallot repair, and arterial switch operations [17]. However, they also noted that in children under six, methadone use was associated with longer hospital stays and higher maximum pain scores, suggesting a need for age-specific protocols. In a separate cohort, Ye et al. conducted a retrospective cohort study of 78 adolescents undergoing posterior spinal fusion and found that those who received methadone both before and after surgery used 45% less opioid over 72 h (2.2 vs. 4.0 mg/kg MME, *p* < 0.001) compared to those managed with hydromorphone PCA alone, with no significant difference in pain scores (~4/10, *p* = 0.34) [33].

### 4.1. Dosing Strategies: Single vs. Multiple Doses

While single-dose intraoperative methadone is common in many surgical protocols, evidence suggests that a multidose approach may offer more consistent analgesia, especially in pediatric patients. Sadhasivam et al. conducted a prospective cohort study of children undergoing posterior spinal fusion, using a novel perioperative multidose methadone strategy and pharmacokinetic analysis. They reported mean peak plasma methadone concentrations of 31.3 ± 10.7 ng/mL and trough levels of 10.2 ± 3.7 ng/mL, well below the typical toxicity threshold (>100 ng/mL). Pain control was effective, with average pain scores of 2.9 ± 0.9 at 24 h, and postoperative opioid requirements were reduced by 60% compared to historical controls (0.24 ± 0.07 mg/kg vs. 0.61 ± 0.13 mg/kg morphine equivalents; *p* < 0.001). Notably, no significant QTc prolongation or respiratory depression was observed in any patient [39]. Murphy and Szokol (2019) conducted a comprehensive clinical focus review and pharmacokinetic modeling analysis, highlighting that single intraoperative methadone doses of 0.1–0.3 mg/kg (or ≥20 mg total) produce 24–36 h of analgesia, reduce 72 h postoperative opioid consumption by 40–60% (*p* < 0.0001), and markedly lower pain scores (in 21 of 27 measured intervals; *p* = 0.001–<0.0001) [9]. For pediatric care, where variability in drug metabolism is high, spreading the dose over time may offer safer and more reliable outcomes.

### 4.2. Safety Considerations: QTc and Variability

Despite its clinical advantages, methadone’s long and variable half-life raises important safety concerns. Prolonged QTc intervals are a known risk, and while rare, they can lead to torsades de pointes, a potentially fatal arrhythmia. Boisvert-Plante et al. cautioned that methadone’s delayed clearance, particularly in younger children or those with comorbidities, warrants routine ECG monitoring when used perioperatively [40]. Brown et al. reviewed methadone’s use in both chronic and perioperative settings, noting that its benefits are well-established, but that careful titration is crucial to avoid sedation, respiratory depression, and cardiac complications [41]. Parikh et al. (2019) further highlighted that pediatric patients are especially vulnerable to opioid-related adverse effects, making age- and weight-specific dosing protocols essential when methadone is used [42]. For instance, they cite standard-of-care dosing recommendations such as an initial IV dose of 0.05 mg/kg in children under 50 kg, with maximum single doses of 1–2 mg, stressing the need for precise titration to avoid adverse outcomes. Additional risk factors for QTc prolongation include concomitant use of other QTc-prolonging medications (e.g., ondansetron, macrolide antibiotics, certain antidepressants), and electrolyte disturbances such as hypokalemia, hypomagnesemia, and hypocalcemia. Awareness and monitoring of these factors, along with routine ECGs, can help mitigate the risk of cardiac complications in pediatric patients receiving perioperative methadone [9,41].

### 4.3. Practical Methadone Protocol in Pediatric Perioperative Care

**Figure 1:**
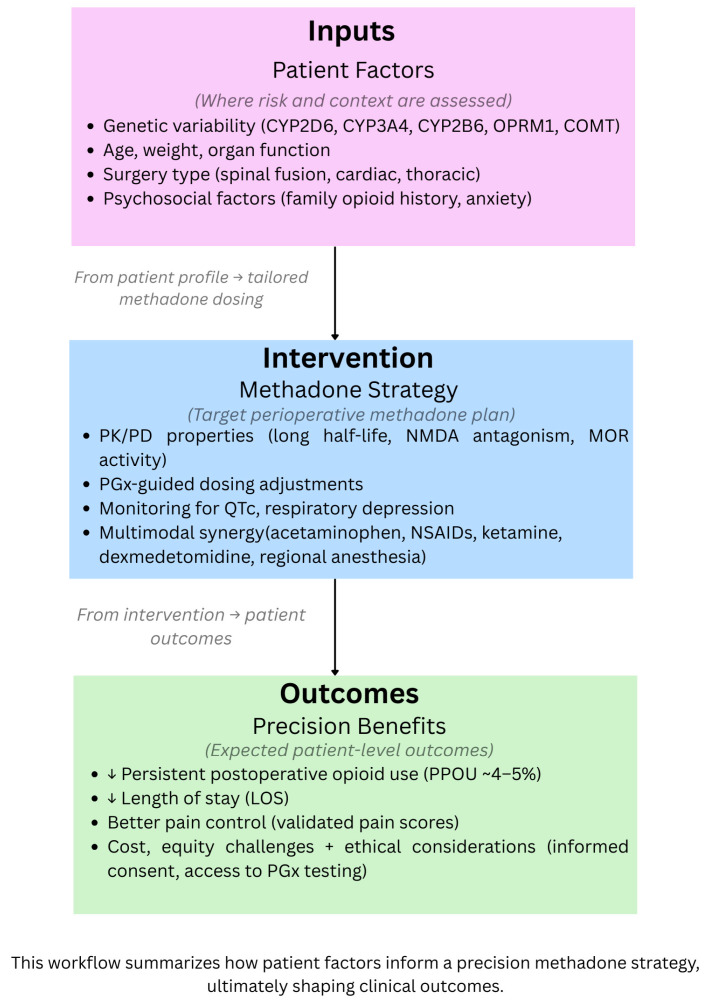
Precision Opioid Strategies in Pediatric Perioperative Care: Integrating Methadone and Pharmacogenomics.

A practical approach to perioperative methadone use in children begins with careful patient selection, prioritizing surgeries associated with high postoperative opioid requirements such as congenital heart surgery, major spine procedures, and thoracic reconstruction, where methadone has consistently reduced opioid consumption and improved recovery trajectories [17,18,32,33,39]. A single intraoperative dose of 0.1–0.2 mg/kg IV is commonly used in pediatric cardiac and orthopedic cohorts, while adolescents undergoing complex spine surgery may benefit from titrated dosing within this range based on anticipated postoperative pain intensity [17,32,33]. Methadone should be administered early during anesthesia induction to leverage its long half-life, NMDA antagonism, and opioid-sparing effects, with additional postoperative doses reserved only for inpatient surgical procedures, to maximize safety given the risk of accumulation. Monitoring should include continuous cardiorespiratory surveillance for at least 24 h, with QTc assessment pre- and post-dose in patients with electrolyte abnormalities, congenital heart disease, interacting medications, or known susceptibility to prolonged repolarization [33,35]. Integration with multimodal analgesia such as scheduled acetaminophen/NSAIDs and regional anesthesia when feasible, maximizes benefit while minimizing opioid exposure. When pharmacogenomic data are available, CYP2D6, CYP3A4, OPRM1, and COMT variants should guide dose adjustment, acknowledging the substantial interpatient variability highlighted across pediatric cohorts [16,18,35]. This streamlined protocol emphasizes safe, individualized methadone administration aligned with current pediatric evidence. Figure 1 illustrates precision opioid strategies in pediatric perioperative care, integrating precision methadone dosing and pharmacogenomic-guided personalized care.

## 5. Pharmacogenetics and Individual Response

One factor that may help explain methadone’s variable response is genetics. Grimsrud et al. explored gene–drug interactions in pediatric burn and surgical patients and found significant interindividual variability in methadone clearance, with a coefficient of variation (CV) exceeding 50% and clearance values ranging from 0.3 to 1.2 L/h/kg [43]. This wide variability underscores the importance of personalized dosing strategies in pediatric populations. Haga et al. argued for broader use of pharmacogenomic testing in pediatrics, suggesting it could enhance safety by enabling more individualized dosing strategies [44]. Integrating genetic information could allow clinicians to tailor methadone administration to each child’s metabolic profile, reducing the likelihood of adverse events while preserving its analgesic benefits.

### 5.1. Emerging Pediatric Evidence and Broader Implications

While much of methadone’s early use in children was based on adult data, a growing body of pediatric-specific studies now supports its inclusion in multimodal analgesia. For example, Sadhasivam et al. evaluated a multidose methadone strategy in 47 children undergoing various surgeries and found stable plasma levels, effective analgesia, and minimal adverse effects including no significant QTc prolongation [39]. Martin et al. conducted a randomized controlled trial with 59 adolescents undergoing posterior spinal fusion and reported significantly lower pain scores (3.4 vs. 5.1 at 12 h) and 30% less opioid rescue use in the methadone group [36]. Blasiole et al. retrospectively reviewed 287 children undergoing cardiac surgery and found a significant reduction in opioid use on the day of surgery (0.3 vs. 0.5 MME/kg, *p* = 0.005) with no increase in adverse events [17]. Chen et al. (2023) further supported methadone’s benefits in pediatric postoperative care, reporting more stable pain scores (±0.6 vs. ±1.5) and a 25% reduction in total opioid use over 48 h compared to short-acting opioids [7]. Together, these findings support methadone’s broader role in pediatric care, suggesting that safe, pharmacogenetically informed dosing may optimize perioperative pain control and outcomes.

### 5.2. Pharmacogenomics

Pharmacogenomics (PGx) offers a detail-oriented approach in prescribing opioids by considering genetic variability for drug metabolism through cytochrome P450 enzymes [45]. It helps refine dosing, reduce adverse events, and improve pain control [42]. This approach is especially relevant in children, where developmental changes and limited clinical study data on ideal dosing [42]. PGX is particularly valuable in pediatrics, where dosing and pharmacokinetics are made complex due to developmental changes and limited clinical trial data [44,46]. Without PGx guidance, patients face higher risks of treatment failure and PPOU. The most clinically relevant enzymes for opioid metabolism are CYP2D6, CYP2B6, and CYP3A4 [44,45]. CYP2D6 metabolizes several opioids, including oxycodone and codeine [44,45]. Codeine depends entirely on CYP2D6 for conversion to morphine, which drives its analgesic effect [47]. Genetic polymorphisms classify patients as poor, intermediate, normal, or ultrarapid metabolizers, each with direct implications for efficacy and safety when using CYP2D6-dependent opioids [48]. Chidambaran et al. (2017) emphasized that these variations lead to serious adverse events for pediatric patients if not accounted for when prescribing, like respiratory depression in up to 41% of patients [47]. They referenced another study to emphasize the importance of PGx, that 10 out of every 100 Caucasians are PMs, whereas 29 out of 100 Ethiopians are UMs [47]. Ethnicity is a key factor of consideration for PGx implementation, in order to avoid under- and over-treatment based on generalized assumptions for dosing [47,49]. Allele frequencies for CYP enzymes vary across subpopulations, with NMs dominating at about 43–67% of the global population [49]. UMs convert codeine into morphine too quickly, which poses a risk for respiratory depression, whereas PMs may result in no analgesic effect and have lower concentrations of active metabolite in their plasma [49]. Wong et al. (2022) noted the same UM variation may influence oxycodone breakdown, as it is active in its parent form via the μ-opioid it partially converts into oxymorphone via CYP2D6 [49].

Aruldhas et al. (2023) ran a prospective pilot study involving 89 pediatric patients (35 pectus excavatum repair and 54 spinal fusion surgery patients), finding higher maximum pain scores, by 2.94, in those whose OPRM1 genes had the recessive single nucleotide polymorphism (SNP), rs3192723 [6]. This SNP has been minimally studied but has a lower response than wild type to methadone (*p* < 0.05). Their postoperative pain was reported on NRSof 0–10, and 59 of said 89 patients required a rescue dose of opioid medication, 77.6% being hydromorphone and 18.8% hydrocodone, potent and active metabolites [6]. These findings support using PGx-educated prescribing to reduce suboptimal pain management, leading away from possible PPOU, through identifying genetic markers [6].

Targeted CYP2D6 genotyping is currently the most cost-efficient and clinically actionable pharmacogenetic test for perioperative opioid personalization in children [48,50]. Data from the CYP2D6-guided opioid prescribing program at Cincinnati Children’s Hospital (Ramsey et al., 2023) demonstrate that single-gene CYP2D6 testing reduces the use of high-risk opioids (e.g., codeine, tramadol), lowers adverse drug events, and improves prescriber confidence [50]. CYP2D6 genotype-to-phenotype translation is reliable, inexpensive relative to panel testing, and integrates well into existing clinical workflows through electronic health record alerts. For these reasons, currently CYP2D6 remains the most practical and cost-effective first-line PGx test for pediatric perioperative care, with other institutions able to replicate this approach using EHR-based Best Practice Advisories [50].

CYP3A4 plays a major role in metabolizing methadone and fentanyl [6,47,51]. It is highly inducible and a key source of drug interactions, which adds complexity to methadone pharmacokinetics [43,52,53,54]. Methadone metabolism is stereoselective, with CYP2B6 preferentially clearing S-methadone and performing most N-demethylation [7,48]. Enzyme variants influence this process: CYP2B61 and CYP2B64 increase methadone clearance, while *6 and *18 reduce it, leading to higher drug exposure [48]. Across 38 CYP-metabolized medications, CYP3A4 served as the primary enzyme for 5 of 6 opioids, compared with CYP2D6′s role in 3, highlighting CYP3A4′s dominant contribution to hepatic opioid clearance [52]. UGT2B7 supports morphine glucuronidation, producing morphine-6-glucuronide (analgesic) and morphine-3-glucuronide (inactive and potentially neurotoxic at high levels) [49,52]. Disruptions in this pathway can cause drug accumulation and toxicity, especially in children and in those with impaired hepatic function [43,48,49]. Reizine et al. demonstrated that CYP2D6 status influenced the needs of oncology patients for opioids, where IM/PM patients often required later-line opioid prescriptions like morphine and hydromorphone, which do not require CYP2D6 metabolism (OR = 3.3; 95% CI, 1.1–9.8; *p* = 0.03) [52]. In multivariable regression analyses, IM/PM were found to be five times more likely to require an intervention or hospital encounter than NMs for pain (odds ratio [OR], 5.4; confidence interval [CI], 1.2–23.6; *p* = 0.03) [52]. While this evidence comes from adult oncology patients, the underlying CYP2D6 biology is shared across age groups, making the findings mechanistically relevant to children. Pediatric data remain limited, but the pattern supports using genotype information to avoid under-treatment and improve postoperative pain control [52]. Even with these compelling findings, implementation across pediatric institutions remains uneven due to the lack of robust data for diverse patient populations [43].

Representing the Sanford Children’s Genomic Medicine Consortium, Gregornik et al. (2020) showcased a large gap: while over 75 drug-gene associations have been identified by the FDA, only a small portion of those have been supported for pediatric use [53]. The Clinical Pharmacogenetics Implementation Consortium (CPIC) has published a total of 26 gene-drug guidelines, to which 4 concern the analgesics codeine, tramadol, oxycodone, and celecoxib [54]. They emphasized that extrapolating adult data does not provide enough for pediatric use, as enzyme expression changes with age, requiring age-specific PGx guidance [53,54]. Aside from genotype, ontogeny has a major role in the drug-gene relationship for pediatric patients. It complicates PGx implementation due to the maturation of drug metabolizer enzymes (like CYP enzymes), and therefore change in functioning ability, over time. This suggests that age-dependent opioid dosing measures should be implemented [53,54].

Aka et al. evaluated CYP-associated drug metabolism in children and contended that the lack of pediatric-specific data to reference impedes safe clinical translation [54]. Of 41 distinct CYP-actionable drugs, only 10 had abundant data concerning 500 pediatric patients on average over 1 year of exposure to the medications [54]. These CYP-associated drugs include oxycodone, codeine, ondansetron, omeprazole, lansoprazole, sertraline, amitriptyline, citalopram, risperidone, and escitalopram. They support CYP2D6 and CYP3A4 genotyping before elective procedures involving opioid utilization. COMT and OPRM1 variants are non-metabolic genes but still contribute to pain perception and sensitivity regarding opioids and are now included in CPIC guidelines for select therapies [48]. OPRM1 variant A118G may decrease opioid binding affinity and analgesia, but does influence post-operative opioid requirements, with evidence for about a 10% increase in morphine dose requirements with patients carrying at least one copy of the gene [6]. COMT Val158Met concerns dopamine metabolism, influencing treatment success and pain response [48].

Finally, Chen et al. differentiated short-acting versus long-acting opioids in pediatric postoperative care and emphasized the need for individualized pain management strategies, including PGx, to improve care and minimize risks [7]. For every 20 out of 100 adolescents undergoing substantial surgeries, insufficient postoperative pain management is a major risk factor for chronic postsurgical pain, which occurs in about 36% of adolescents undergoing posterior spinal fusion, based on a prospective cohort study of 133 patients by Chidambaran et al. [55]. Methadone has a role in blocking the reuptake of neurotransmitters, norepinephrine and serotonin, reducing the likelihood of developing postsurgical pain [7]. With the incorporation of a methadone-only protocol, 122 pediatric spinal fusion patients had 45% lower opioid consumption, reduced length of stay, and lower opioid-related side effects than those receiving only hydromorphone [7]. Their expert review highlighted that while PGx is not yet standard care, growing evidence supports pharmacogenomic testing as a part of future routine practice [7]. Key pharmacogenomic variants relevant to methadone and opioid metabolism in pediatric perioperative care are summarized in Table 1.

Clinical Implications: Based on the findings in this study, several practical considerations can help guide clinical use. Routine monitoring must involve vital signs, sedation scales, standardized pain scores [6], and ECG for QTc [40] in order to identify early toxicity or insufficient analgesia. Weight-based pharmacokinetics, single versus repeated dosing [9,39], and PGx-informed variations in CYP2D6 or CYP3A4 metabolism [45] can all be taken into account when adjusting doses. To optimize medication dosage and reduce adverse effects [7], preoperative PGx screening can identify children with high-risk variants affecting opioid sensitivity, slow metabolism and QT prolongation risk. PGx testing could facilitate safer, individualized pain management and improve perioperative workflows when incorporated into pre-operative evaluations for high-risk surgeries.

### 5.3. Barriers and Ethical Considerations

The perioperative use of methadone in pediatric surgical care, while promising for sustained analgesia and opioid-sparing effects, faces several clinical, ethical, and systemic barriers. These challenges span pharmacologic safety, genetic variability, infrastructure limitations, health equity, and the ethical balance between personalized pain relief and long-term harm prevention with additional cost in limited populations.

Safety and Pharmacologic Complexity: Methadone’s long and highly variable half-life in children, reported from 8 to more than 59 h depending on age, CYP enzyme activity, and comorbidities, increases the risk of dose accumulation and respiratory depression. Respiratory depression rates have reached 22.6 percent after a single intraoperative dose in children, which contributes to provider reluctance even though newer multi-dose protocols have demonstrated more stable plasma concentrations below the 100 ng/mL toxicity threshold [39]. QTc prolongation represents another major concern. The S-enantiomer is primarily responsible for this effect and, in rare cases, may precipitate torsades de pointes [56]. Pediatric studies usually report minimal QTc changes (<±30 ms) without clinical arrhythmias, but these shifts are not well studied in large pediatric studies including children undergoing cardiac surgeries. Rises in postoperative alpha-1 acid glycoprotein (AAG) may protect against methadone’s adverse effects. Increased AAG binds methadone and lowers its free fraction, complicating dose–response interpretation [6,39]. Children with elevated AAG may paradoxically have less effective analgesia despite normal measured total levels, yet free methadone is rarely assessed in clinical practice.

Variability in Metabolism and Monitoring Burdens: Wide interindividual differences in methadone metabolism, driven by CYP2B6, CYP3A4, and CYP2D6 activity, create dosing uncertainty, especially in younger children with immature enzymatic pathways [7]. Postoperative increases in AAG and unpredictable enzyme expression further challenge fixed-dose strategies and highlight the need for precision dosing tools that many pediatric centers lack.

Methadone’s delayed clearance complicates discharge planning. In a PICU weaning protocol, tapering reduced the number of children discharged on methadone by more than 50 percent, but rates of mild to moderate withdrawal increased, underscoring the ethical need for shared decision-making and clear communication with families during opioid weaning [57]. Clinicians must weigh the benefits of structured inpatient tapering against the distress of withdrawal, particularly in settings with limited post-discharge monitoring.

Infrastructure constraints add another layer. Methadone protocols require ECG monitoring, opioid stewardship pathways, and clinician education in both methadone pharmacology and pharmacogenomics. Many providers lack formal training in interpreting genotype results or adjusting doses based on metabolic variability.

Pharmacogenomic Challenges: Although pharmacogenomics could personalize methadone dosing and reduce adverse events, several practical and ethical issues limit routine use. CYP2D6 genotyping may help identify UMs or PMs, but pediatric PGx guidelines remain sparse. Most CPIC dosing recommendations are extrapolated from adults and do not account for age-specific enzyme maturation or developmental differences in drug handling [50].

Other obstacles include high cost, slow turnaround times of 1 to 3 weeks, and limited reimbursement in perioperative pediatrics. Consent for genetic testing is often rushed or inconsistently obtained, which complicates ethical implementation. Many hospitals also lack integrated clinical decision support systems to convert genotype data into actionable dosing guidance [16].

Health equity further complicates PGx adoption. CYP2D6 allele frequencies vary widely across populations, for example, up to 29 percent of Ethiopians are UM when compared with fewer than 2 percent of East Asians [7,16,50]. Implementing PGx-guided protocols without attention to population-specific variation and equitable access may widen disparities in personalized pain management.

Pediatric-Specific Ethical Responsibilities: Ethical considerations are heightened in pediatric practice, where caregivers must make complex decisions about medications with potential long-term neurodevelopmental implications [58]. Shared decision-making is essential when considering methadone, an opioid with both substantial analgesic benefit and risks of respiratory or cardiac complications. Caregivers should receive clear explanations of methadone’s rationale, expected monitoring needs, and plans for tapering or weaning after surgery. Institutions should provide interpreter support, culturally appropriate communication tools, and literacy-adapted materials to ensure equitable understanding [56,57].

Methadone must also be situated within a broader opioid stewardship framework. Pediatric anesthesiologists and surgeons must balance immediate opioid analgesia with long-term safety. When supported by pharmacogenomics, transparent consent processes, and reliable monitoring systems, methadone can help bridge these goals. Without adequate safeguards, however, even well-designed opioid-sparing protocols risk unintended harm.

### 5.4. Future Directions

Despite growing interest in methadone for perioperative pain management and pharmacogenomics-guided therapy, pediatric evidence remains limited. In a large retrospective cohort by Blasiole et al., only 67 (23%) of 287 pediatric cardiac surgery patients received intraoperative methadone [17], with its use outside spinal and cardiothoracic procedures scarcely reported. Similarly, PGx testing in children is inconsistent compared to adults. Most studies are small and observational; for example, Sadhasivam et al. evaluated 38 adolescents undergoing spinal fusion [39]. These gaps highlight the urgent need for well-designed, large-scale RCTs assessing the safety and efficacy of PGx-guided methadone protocols, including comparisons with fentanyl in pediatric orthopedic surgery.

Personalized pain management is increasingly driven by metabolomics and PGx, which identify biomarkers influencing opioid metabolism, pain sensitivity, and response. Variations in CYP2D6, COMT, OPRM1, and ABCB1 are notable examples [59,60,61,62]. CYP2D6 affects the metabolism of codeine, tramadol, and dihydrocodeine [60,62,63]; COMT influences pain perception and sympathetic tone through catecholamine breakdown [64,65,66]; ABCB1 modulates opioid transport across the blood–brain barrier [64,65,66]; and OPRM1 encodes the mu-opioid receptor, affecting analgesic efficacy [62]. Incorporating these biomarkers may improve methadone’s efficacy and safety in children.

The CPIC provides peer-reviewed PGx guidelines, but most are based on adult data. Of 28 CPIC guidelines, only a few address pediatric-relevant drugs (e.g., codeine, voriconazole, SSRIs, thiopurines), while methadone, hydrocodone, and oxycodone lack pediatric-specific recommendations [50,67]. This is a major limitation given ontogeny-related shifts in enzyme expression- CYP3A4 and CYP2D6 are low at birth and increase with age [68,69].

Despite limited pediatric guidance, early implementation efforts show promise. Programs at St. Jude Children’s Research Hospital, Children’s Mercy Kansas City, and the University of Florida’s GatorPGx demonstrate feasibility and clinical impact [70]. At St. Jude, clinicians adhered to 92% of 4783 clinical decision support alerts across 12 genes and 60 drugs in a preemptive PGx program for critically ill children [71].

PGx can also enhance Enhanced Recovery After Surgery (ERAS) and Procedure-Specific Postoperative Pain Management (PPAP) through individualized, opioid-sparing strategies. ERAS protocols may avoid codeine in CYP2D6 poor or ultra-rapid metabolizers, while favoring methadone for its CYP2D6-independent metabolism [48,72]. Moreover, PGx may identify children at risk for chronic pain or opioid dependence, as seen with COMT Val158Met and OPRM1 A118G variants [73,74]. To fully realize these benefits, multicenter RCTs are essential to validate and standardize PGx-guided methadone use, particularly in pediatric spine, cardiac, and major abdominal surgeries.

Integration of artificial intelligence (AI) with PGx further strengthens predictive pain management. AI models like LSTM-based opioid misuse predictors and GPT-4-powered retrieval-augmented generation (RAG) systems for PGx interpretation demonstrate emerging clinical utility [75,76]. Complementary real-world registry studies at high-volume academic centers with perioperative data infrastructure and anesthesiology outcomes research can expedite evidence generation and guide trial design [77]. Collectively, these approaches are vital for advancing personalized perioperative opioid strategies in children.

## 6. Conclusions

Precision opioid strategies are urgently needed in pediatric perioperative care, where both inadequate pain control and opioid overexposure contribute to chronic postsurgical pain, delayed recovery, and long-term dependence. Methadone’s multimodal analgesia, NMDA antagonism, long half-life, and low cost make it a compelling option, particularly when personalized via pharmacogenomics. PGx-informed methadone protocols could reduce the risk of PPOU, which affects approximately 4–5% of opioid-naïve adolescents after surgery despite current multimodal regimens. Pediatric patients have dynamic pharmacokinetics and genetic variability, such as CYP2D6 polymorphisms, which make one-size-fits-all opioid strategies ineffective. Integrating PGx testing into perioperative pathways, particularly for high-risk surgeries like spinal fusions or thoracic procedures, may improve analgesic efficacy while minimizing adverse events such as respiratory depression or QTc prolongation. Clinical pilots have already shown feasibility, but broader adoption requires investment in safety monitoring, provider education, and family counseling on genetic testing. Multidisciplinary teams comprising anesthesiologists, pharmacists, geneticists, and ethicists should lead the integration of precision analgesia into routine care. Efforts must also prioritize equity, ensuring PGx testing is validated across diverse pediatric populations, where allele frequencies differ significantly by ancestry. As opioid stewardship becomes a clinical and moral imperative, integrating methadone and PGx into perioperative care represents the next frontier in safe, effective, and individualized pediatric pain management.

## Figures and Tables

**Table 1:** Pharmacogenomic Variants Relevant to Methadone and Opioid Use in Pediatric Perioperative Care.

Gene/Enzyme	Variant(s)	Role in Opioid PK/PD	Clinical Implications	Pediatric Evidence/Notes
CYP2D6	PM, IM, NM, UM phenotypes	Converts codeine → morphine; affects oxycodone (→ oxymorphone)	PMs: no analgesia; UMs: rapid morphine conversion → toxicity/respiratory depression	41% pediatric patients with adverse effects if unaccounted; ethnic variation (10% Caucasian PM, 29% Ethiopian UM) [47]; Cincinnati Children’s protocol reduced adverse events [50]
CYP2B6	*1, *4 ↑ clearance; *6, *18 ↓ clearance	Major methadone N-demethylation; stereoselective for S-methadone	Alters methadone plasma levels → risk of under- or over-treatment	Pediatric dosing variability; risk of treatment failure if ignored [38]
CYP3A4	Polymorphisms less common; enzyme highly inducible	Metabolizes methadone and fentanyl; dominant clearance enzyme	Induction and drug interactions complicate methadone PK	Ontogeny affects CYP3A4 activity in children; dosing adjustment required [47,51]
UGT2B7	—	Morphine glucuronidation → M6G (analgesic), M3G (inactive/neurotoxic)	Disruption → accumulation, neurotoxicity	Especially relevant in neonates and hepatic immaturity [49]
OPRM1	rs3192723; A118G	μ-opioid receptor binding and response	rs3192723 → higher pain scores, lower methadone response; A118G → ↓ binding affinity, ↑ morphine needs	Aruldhas 2023: NRS +2.94 pain; ↑ rescue opioid use in carriers [6]
COMT	Val158Met	Dopamine metabolism; modulates pain sensitivity	Alters analgesic response and treatment success	CPIC-guided; affects opioid requirement in pediatric surgery [48]

Arrows indicate the direction of change in drug clearance (↑ increased clearance, ↓ decreased clearance).

## Data Availability

The original contributions presented in this study are included in the article. Further inquiries can be directed to the corresponding author.
